# Plasma-Generated OH Radical Production for Analyzing Three-Dimensional Structure in Protein Therapeutics

**DOI:** 10.1038/s41598-017-13371-7

**Published:** 2017-10-11

**Authors:** Benjamin B. Minkoff, Joshua M. Blatz, Faraz A. Choudhury, Daniel Benjamin, J. Leon Shohet, Michael R. Sussman

**Affiliations:** 1 Department of Biochemistry, University of Wisconsin-Madison, Madison, WI USA; 2 Department of Electrical and Computer Engineering, University of Wisconsin-Madison, Madison, WI USA

## Abstract

Protein three-dimensional structure dynamically changes in solution depending on the presence of ligands and interacting proteins. Methods for detecting these changes in protein conformation include ‘protein footprinting,’ using mass spectrometry. We describe herein a new technique, PLIMB (Plasma Induced Modification of Biomolecules), that generates µs bursts of hydroxyl radicals from water, to measure changes in protein structure via altered solvent accessibility of amino acid side chains. PLIMB was first benchmarked with model compounds, and then applied to a biological problem, i.e., ligand (EGF) induced changes in the conformation of the external (ecto) domain of Epidermal Growth Factor Receptor (EGFR). Regions in which oxidation decreased upon adding EGF fall along the dimerization interface, consistent with models derived from crystal structures. These results demonstrate that plasma-generated hydroxyl radicals from water can be used to map protein conformational changes, and provide a readily accessible means of studying protein structure in solution.

## Introduction

The three-dimensional structure of proteins can be studied in solution using various methods of covalent and noncovalent labeling in which the readout is accomplished by mass spectrometry (MS). These procedures, collectively known as protein ‘footprinting’, all involve measuring the solvent accessibility of either the peptide backbone (hydrogen deuterium exchange) or amino acid side chains (covalent modification and cross linking reagents)^1^. After labeling, proteins are analyzed using tandem MS and linkages or adducts are quantitatively mapped to specific regions, revealing areas and degrees of intermolecular interactions or solvent accessibility. The emergence of the quickly expanding protein therapeutics industry^2^ has generated a need for easy, sensitive, and reproducible methods for detecting changes in protein conformation, as part of the rigorous quality control process required for clinical use and for quickly detecting the regions of proteins involved in their binding to ligands and/or protein-protein interactions.

One of the more prevalent means of noncovalent protein footprinting is Hydrogen Deuterium Exchange (HDX), which utilizes deuterium exchanged with hydrogen atoms along the peptide backbone to measure solvent accessibility^3^. HDX is so widely used that there now exist automated systems to improve throughput and reproducibility However, HDX is not technically easy to perform: time-sensitive back-exchange between deuterium and naturally abundant hydrogen occurs, so the technique must be rapidly performed at very low temperatures. In addition, HDX relies upon acidic conditions for quenching the exchange reaction, necessitating the use of the non-specific protease, pepsin, for digestion. Finally, the solvent accessibility information obtained is tied to the peptide backbone, rather than to amino acid side chains and while important, may not provide a complete picture of the protein’s three-dimensional state.

Protein footprinting performed with chemical reagents, while similar in principle to HDX, results in covalent modification of amino acid side chains. A number of reagents for this purpose are available, such as EDC/GEE for carboxyl labeling^4^, trypsin-based methods for detecting changes in accessibility to proteolytic enzymes^5^, highly reactive carbenes^6^, and hydroxyl radicals^1^. Hydroxyl radical footprinting (HRF) for protein structure analysis is mainly performed with two well-known methods of generating hydroxyl radicals: fast photochemical oxidation of proteins (FPOP)^7^ and synchrotron-based radiolysis of water to form hydroxyl radicals^8^. It should also be noted that electrical discharge has previously been used for radical generation, but it has been limited to electrospray devices and has not been widely adopted^9,10^.

Whereas synchrotron radiation generates radicals directly from water, FPOP uses a 248nm-excimer laser to generate radicals from hydrogen peroxide added to a sample prior to exposure^7^. Though both techniques can be used for labeling experiments, each has drawbacks that limit widespread adoption. Synchrotron experiments generate hydroxyl radicals via radiolysis of water and thus do not involve added chemicals^8^, but they nonetheless require access to one of the handful of major synchrotron radiation source facilities and with the current technologies labeling occurs on ms timescale^8,9^. While FPOP can be employed on a benchtop scale, the technique requires hydrogen peroxide and exposure to a 248 nm laser, within the absorption range of aromatic residues. Additionally, there is evidence that an FPOP-induced oxidative environment may occur over a millisecond, rather than a microsecond timescale^11^, and this longer-term exposure could potentially perturb protein structure, particularly in proteins that are rapidly altering their structure in solution.

In this report, we describe a technique termed PLIMB (Plasma-Induced Modification of Biomolecules), in which HRF is performed using a plasma to generate µs pulses of hydroxyl radicals, from water, in solution. After calibrating this technology with free methionine, we then studied its use with bovine serum albumin (BSA), to benchmark PLIMB with a structured model protein. For a new application with a protein of importance in basic biological research as well as in clinical settings, PLIMB was then used to analyze which amino acids are involved in the conformational changes that occur when epidermal growth factor (EGF) is added to epidermal growth factor receptor (EGFR). EGFR is particularly important as a therapeutic target for cancer treatments^12^. There is thus great interest in obtaining a firm handle on its three-dimensional structures under different conditions in solution. While a crystal structure of full-length EGFR has yet to be solved, various domains, including the externally located (ecto) domain that binds EGF on the outside of the cell, have been crystallized and had structures solved at high resolution. Given the great interest in this protein as both a diagnostic and therapeutic tool, we have tested our method using plasma-generated hydroxyl radicals to perform footprinting with the ecto domain of EFGR before and after the ligand, EGF, is bound.

## Results

### PLIMB generates µs pulses of hydroxyl radicals and labels free methionine in solution

**Figure 1:**
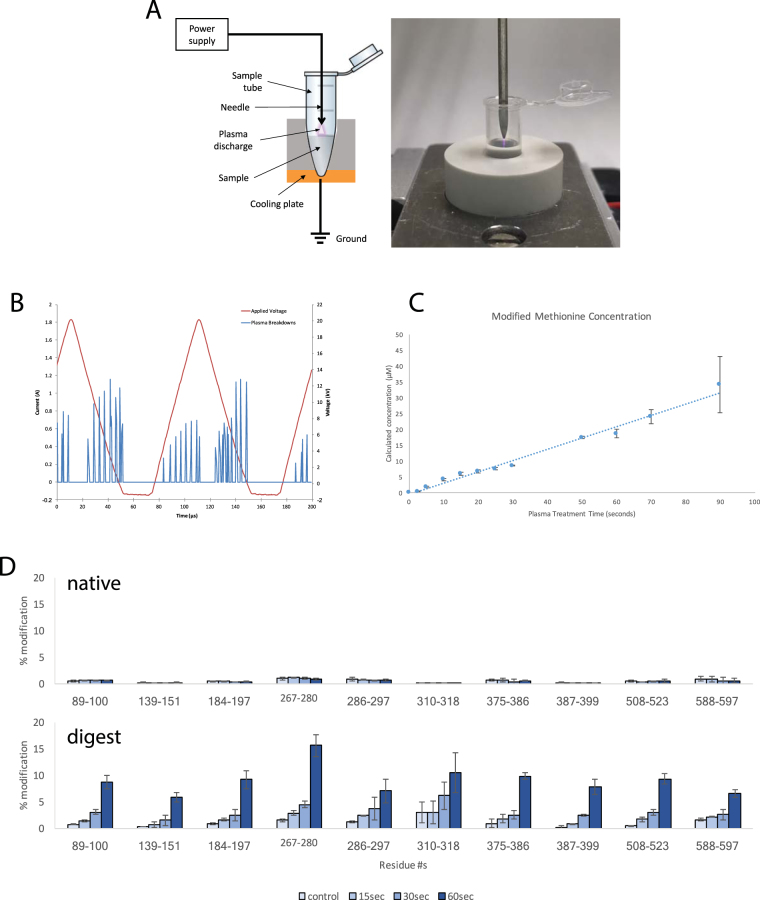
Microsecond bursts of radicals modify free methionine and BSA in a dose dependent fashion. (**A**) Left, schematic of sample and instrument. Right, photograph demonstrating treatment in progress. (**B**) Graph demonstrating µs breakdowns to produce µs bursts of hydroxyl radicals. (**C**) Production of oxidized methionine with increasing plasma treatment time. For each data point, n = 3 and error bars are ± standard deviation. (**D**) Modification observed on native (above) and digested (below) BSA with increasing plasma treatment times. Regions shown for comparison have been selected; full data in Fig. S3. For each bar, n = 3 and error bars are ± standard deviation.

It is known that plasma can be used to generate hydroxyl radicals in water, but the literature surrounding the use of such radicals for protein footprinting is sparse^10^. In this report, we describe a technique geared towards biological experiments in which hydroxyl radicals, generated from plasma on a µs timescale, were used to label proteins dissolved in lightly buffered aqueous solutions, for solvent accessibility measurements. To do this, an instrument was built that generates a plasma, which is directed through a liquid sample, using a dielectric-barrier discharge (Fig. 1A). Figure 1A, left, shows a diagram of the plasma setup. A sample is placed above a grounded cooling plate and needle is fixed approximately 1 mm above the surface of the sample. A time-varying high voltage signal is applied to it which then creates a plasma as seen in Fig. 1A, right. The system can be operated over a range of high voltages from 1–31 kV, and a range of frequencies from 0 to 15 kHz. In between pulses, there is sufficient time, as reported in the literature, for radicals to recombine and dissipate (Fig. 1B)^13^.

The amino acid methionine is readily converted to methionine sulfoxide after oxidation and is often used as a scavenger for hydroxyl radical experiments. Thus, in our preliminary experiments to calibrate the instrument, an aqueous buffered solution of methionine was treated with PLIMB for various periods of time. MS analysis revealed that, over a period of 90 seconds, methionine is nearly stoichometrically converted to methionine sulfoxide, proportional to the plasma exposure time (Fig. 1C). This observation is consistent with a system that generates a constant flux of hydroxyl radicals per unit time, limited to the brief µs long interval in which the voltage is sufficiently high to create a discharge. Using purified methionine sulfoxide, a concentration curve was made, under the same conditions. Comparing the concentration of methionine sulfoxide generated with plasma dose corrected for the finite pulse width to this curve yielded a minimum hydroxyl radical generation of roughly 350 nmol/sec. Thus, PLIMB generates measureable amounts of hydroxyl radicals, in µs bursts, in a reproducible, dose-dependent fashion from an aqueous buffered solution.

### The protein BSA maintains its structure throughout PLIMB exposure

The results from the experiments with methionine (and other free amino acids, unpublished) in solution suggested that PLIMB could also label proteins in solution. This was tested using Bovine Serum Albumin (BSA), a well-studied ~66 kDa protein with a known tertiary structure. BSA was dissolved in phosphate buffered saline at a concentration of 50uM, and treated with PLIMB for up to 60 seconds. We observed dose-dependent modification in multiple discrete regions of the protein, and oxidation on multiple amino acids (Fig. 1D, Table S1), while BSA remained intact (Fig. S3). Within the timeframe used, regions that are exposed to bulk solvent are saturated with oxidation, whereas those that are buried contain less reactive amino acids and exhibit a much slower rate of oxidation at a baseline level that is mainly unaltered during the time course of PLIMB treatment.

Any method using covalent protein footprinting reagents has the potential to perturb the tertiary structure and thus, we tested whether or not PLIMB maintains protein structure throughout exposure by repeating the experiment, but using BSA that has been digested with Trypsin/Lys-C prior to analysis (predigestion). Protein loads, solution buffers, and exposure times were controlled between experiments. If PLIMB is not perturbing the protein’s structure, and the less accessible residues remain buried inside its structure, heavier oxidation should be observed when we denature BSA prior to exposure. Indeed, the data revealed that denaturation via predigestion causes more oxidation to occur on many amino acids in BSA with PLIMB treatment (Fig. 1D, Table S1). Importantly, regions that maintained a baseline level of oxidation when native/undigested BSA was treated now exhibit a dose-dependent increase in their oxidation state, suggesting that although they may have been buried internally in the native undenatured state, upon digestion, they are exposed to bulk solvent and free to react with plasma-generated radicals. Altogether, the results of the BSA experiment are consistent with our hypothesis that PLIMB oxidizes protein in a native state to reveal solvent accessible regions, and that protein structure is maintained during the treatment.

### PLIMB identifies regions involved in EGF-altered EGFR conformation

**Figure 2:**
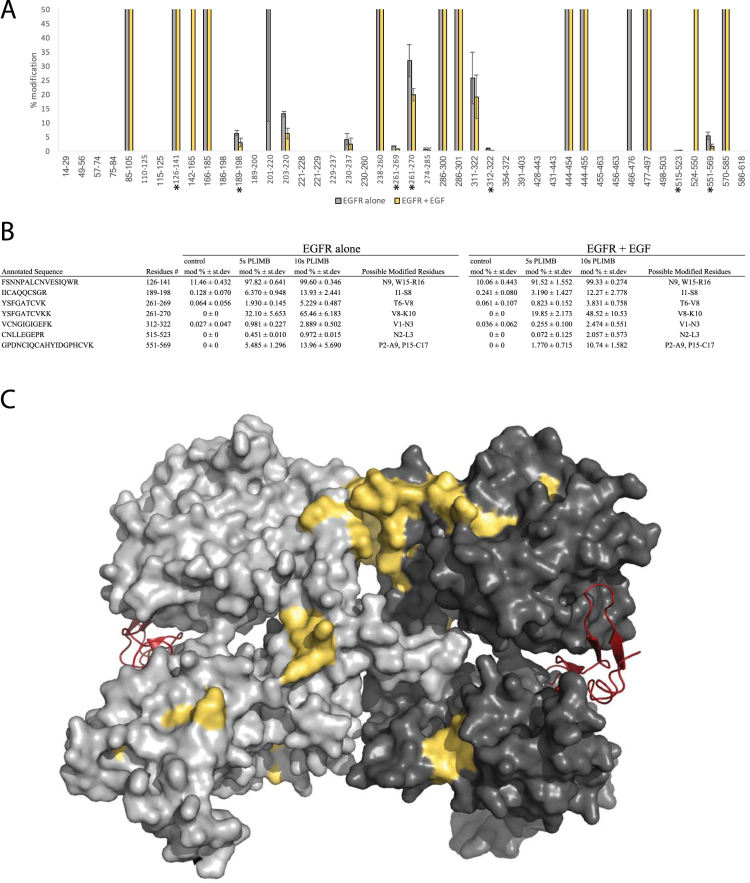
PLIMB identifies regions of EGFR that are decreased in solvent accessibility when EGF is added. (**A**) Modification observed on EGFR alone (grey) and with added EGF (yellow). All data are five seconds of PLIMB treatment. For each bar, n = 3 and error bars are ± standard deviation. Asterisks indicate statistical significance (p < 0.05). (**B**) Table containing modified residues from and information regarding peptides shown in Fig. 2A. (**C**) Space fill model of the EGF-bound and dimerized extracellular domain of human EGFR. Monomers shown in light and dark grey. EGF shown in red, and residues for which oxidation decreased in a statistically significant fashion when EGF is added are colored yellow. Image of 1IVO^15^ created using PyMOL (The PyMOL Molecular Graphics system, Version 1.8, Schrödinger, LLC).

One of the most promising applications of protein footprinting experiments is the ability to map protein-protein interaction domains and other structural features, under a variety of conditions in solution. We next tested PLIMB by labeling epidermal growth factor receptor (EGFR), a protein with implications in many cancers, before and after binding one of its native ligands, epidermal growth factor (EGF). The currently accepted model for *in vivo* EGFR functionality involves 2 molecules of EGF binding to 2 molecules of EGFR, inducing homodimerization and ultimately stimulating a signaling cascade with multiple downstream responses^14^. Using the extracellular (ecto) domain of purified human EGFR and EGF, we mapped structural changes induced by allowing the protein and ligand to interact *in vitro*. A five and ten second PLIMB treatment of µs-long pulses was used, and oxidation was mapped and quantified in amino acids throughout EGFR treated with and without added EGF. After trypsin digestion and MS/MS analysis, we observed that a discrete subset of tryptic peptides showed statistically significant decreases in the plasma-induced oxidation state upon EGF addition (Fig. 2A,B, Table S2), suggesting that the ligand reduced their solvent accessibility.

Manually inspecting the database search results and MS/MS spectra of the peptides in regions of changing solvent accessibility allowed us to narrow the changes down to individual or smaller groups of amino acids, and we mapped these to the known crystal structure of the EGF-induced homodimerized extracellular domain of human EGFR^15^ (Fig. 2C, Table S2). Various regions that have previously been implicated in dimerization experienced decreased oxidation. Decreased oxidation clusters occurred spatially at the top of the interface between the EGFR monomers, suggesting we are observing major overall decreases in solvent accessibility of this region, consistent with known properties of the protein and EGF-induced dimerization.

## Discussion

Protein footprinting is an area that offers unique advantages compared with classical structural techniques–the ability to gain structural information on proteins in solution and map conformational changes by tracking solvent accessibility. Having a relatively quick MS/MS readout makes this technology compelling. The high reactivity of hydroxyl radicals with many molecules offers the advantages of stable covalent modification with the vast majority of amino acid side chains, a relatively small chemical adduct, and rapid reaction times^9^.

PLIMB, which is workable on a benchtop, applicable to a range of protein concentrations and sizes, and generates µs bursts of hydroxyl radicals without added chemicals or reagents (Fig. 1A,B) has been developed and the results benchmarked. PLIMB was first tested with free methionine in an aqueous buffered solution. With a concentration of 50 µM, and treatment times up to 90 seconds, saturation of product formation was not achieved, and the appearance of the modified product, methionine sulfoxide, was linear, suggesting that radicals are generated continuously within this time frame (Fig. 1C). Data was acquired using an electrospray ionization time-of-flight mass spectrometer, scanning over a large mass range. Interestingly, the only ‘classical’ methionine and hydroxyl radical product observed was methionine sulfoxide, at +16 m/z. This is in contrast to synchrotron-generated hydroxyl radical chemistry, for which the sulfoxide (+16), sulfone (+32), and aldehyde (−32) products are all observed, although these experiments differed from ours in treatment time (up to 24 minutes) and in using a tripeptide with the composition H-Gly-Met-Gly-OH, rather than free methionine^16^. It may be that the PLIMB treatment times are too short to observe these secondary products, or that the radical generation rate is too slow to produce classical products other than methionine sulfoxide, given differing primary (+16) and secondary (+32 and −32) product formation rates. Constructing a concentration curve using purified methionine sulfoxide revealed that, based on the appearance of methionine sulfoxide over time, PLIMB is generating hydroxyl radicals at a rate of roughly 350 nmol/sec via spaced µs discharge bursts. This is taken to be the minimum value for radical generation, as we cannot rule out unobservable product generation (e.g., products that don’t charge properly during electrospray ionization or exist below the limit of detection) and radical recombination as side reactions that at this time have not been measured.

PLIMB was next used to treat a readily available model protein, bovine serum albumin (BSA), in solution. Two experiments were done with BSA–PLIMB treatment up to 60 seconds with BSA in its native, folded state, and alternatively, with BSA denatured by predigestion with trypsin prior to PLIMB exposure. The results suggest that PLIMB does not perturb protein structure during these exposure times, for several reasons. First, there are regions that exhibit virtually no modification in the native BSA that, when digested, become susceptible to oxidation with PLIMB treatment, such as peptides containing residues 310–318 and 387–399 (Fig. 1D, Table S1). Second, some regions exhibit a baseline level of modification that occurs independently of PLIMB treatment (i.e., there is no dose-dependent modification observed as a result of PLIMB treatment) when BSA is native; however, when BSA is digested prior to treatment, these regions exhibit a dose dependent increase in modification. Thus, digestion releases them from their hydrophobic, buried state so that they can now react with the bulk solvent to become oxidized. Examples of this include peptides containing residues 89–100 and 347–359 (Fig. 1D, Table S1). Altogether, these data suggest that not only does PLIMB modify accessible regions of a protein in a dose-dependent manner, but that the protein’s structure is maintained throughout exposure.

**Figure 3:**
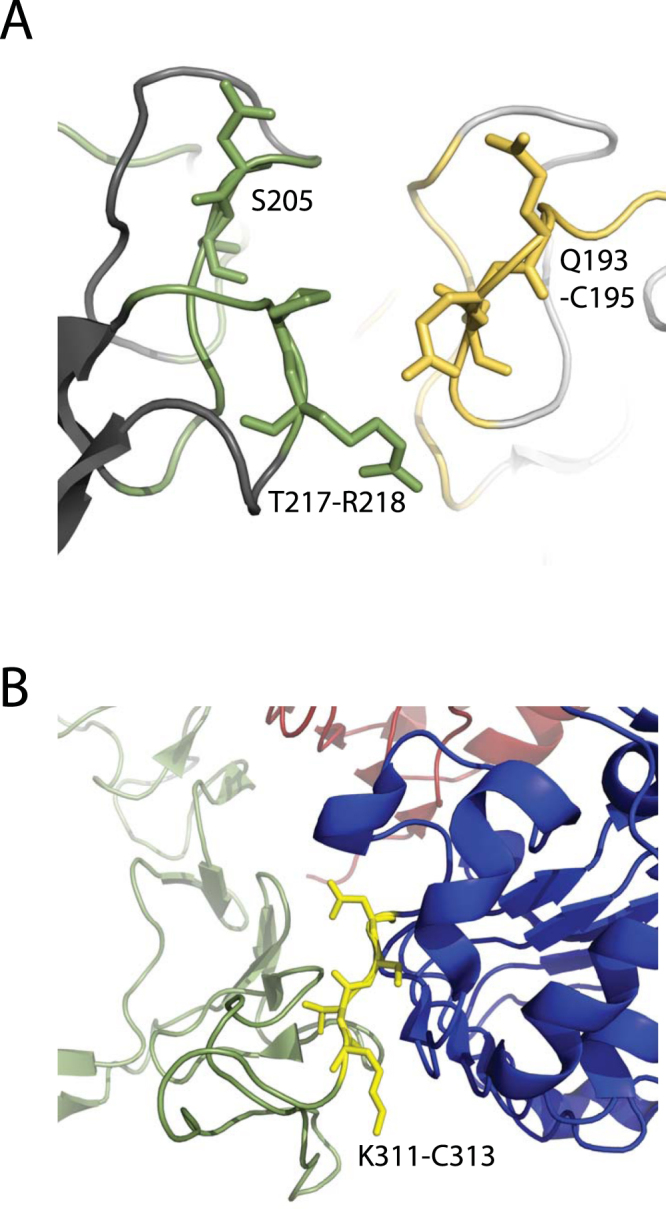
PLIMB identifies regions previously implicated in EGF-induced structural changes. (**A**) Close up view of residues within cluster at top of dimerization interface, shown in the crystal structure. These were identified as decreased in oxidation state when EGF is added to EGFR in solution in the experiments described herein. Green and yellow are the residues on either monomer of the dimer, and residue numbers are labeled. (**B**) Close up view of “hinge region” spanning Domains II and III, for which arrangement changes upon binding EGF. Domain I is shown in red, Domain II green, and Domain III blue. Domain IV is not pictured. Residues for which oxidation decreased upon adding EGF are shown in yellow and labeled. Image of 1IVO^15^ created using PyMOL (The PyMOL Molecular Graphics system, Version 1.8, Schrödinger, LLC).

One of the more promising uses for protein footprinting is to map structural changes under a variety of conditions. We used PLIMB to map EGF-induced structural changes in the extracellular domain of EGFR, and found multiple regions containing residues whose oxidation state is decreased in response to EGF application (Fig. 2A,B). Interestingly, a large spatial cluster of such decreasing oxidation is located directly at the dimerization interface derived from published crystal structures^15,17^ (Fig. 2C). The observation of residues that are modified and decreased in oxidation within the spatial cluster is also consistent with previous findings in experiments on EGFR dimerization. For example, residues Q193-C195 and S205, identified here as decreased in oxidation state when EGF is added to EGFR, make contacts between EGFR monomers in the TGFα-induced dimer^17^ (Fig. 3A), and the region containing residues 261–270 is also involved in similar intermolecular contact.

PLIMB also identified areas distinct from the dimerization interface that have been implicated in intramolecular structural changes upon binding EGF. We observed decreasing oxidation on the region containing residues K311-K322 (Fig. 3B, Table S2). This is consistent with published data–the conformation surrounding K311, known as the hinge region between domains II and III of EGFR, changes as a result of domain II contacting domain III in the EGF-bound crystal structure^15^. PLIMB identifies decreased solvent accessibility in this region of increased intramolecular contact, specifically around residues K311-C313.

Though the active EGF-bound EGFR protein has not been crystallized in its entirety, the structure of the extracellular (ecto) domain of EGFR has been solved^18^. Using this information, we identified decreasing oxidation on residues within EGFR’s domain IV, specifically within the region containing residues G551-K569, which were included in the construct used for solving the structure of monomeric, but not dimerized, EGFR^18^. Examining the structure of the monomeric extracellular EGFR reveals that this region makes contact on one face with domain II when inactivated (Fig. S8), and previous modeling suggests significant contact between domain IV on either monomer within the dimerized complex^18^. Furthermore, the same report demonstrated that a protein with a D563A/H566A/K585A triple mutation exhibits a significant decrease in ability to bind EGF and activate, almost to the same degree as a variant lacking Domain IV. PLIMB identified decreased oxidation at five seconds of treatment, on the region containing D563 and H566, specifically on either side of H566 (Table S2). Thus, the importance of this domain and these residues for EGF binding and activation via dimerization is consistent with the PLIMB data described herein.

Other regions and residues that have not been implicated in dimerization were also revealed to have their oxidation states decreased when EGFR dimerizes. These include localized areas containing residues W140/R141, and W453/K454. We hypothesize that these residues may experience less solvent accessibility due to force exerted through the molecule upon dimerization, leading to a slightly more closed conformation in these areas. For example, W140/R141 lie in proximity to the region containing I190/C191, Q193-C195, and D206/C207, all of which have less oxidation upon dimerization, suggesting there may be a tightening of this region in solution when dimerized. Altogether, the PLIMB derived EGFR data suggest minor differences in solvent accessibility when compared to the structure of EGFR solved by crystallographic techniques, though areas revealed to undergo large conformational changes, and to be important for both EGF binding and activation upon EGF-induced dimerization, are similarly identified using both techniques.

In summary, the techniques currently available for hydroxyl radical protein footprinting (synchrotron radiolysis of water, laser-induced production of hydroxyl radicals from hydrogen peroxide) are powerful, but they have yet to be implemented to a widespread degree. Here, we present PLIMB, a technique that generates µs bursts of hydroxyl radicals from water, for protein footprinting and structural feature determination in solution using an instrument that involves relatively low cost electronic components and is easy to operate. PLIMB was used to map the dimerization of EGF-induced EGFR dimerization, yielding results consistent with published crystal structures and demonstrating its promising utility for quick mapping of protein-protein interactions for applications in the clinical protein therapeutics field, as well as for basic research on the three-dimensional structure of proteins in solution under a wide number of conditions.

## Methods

### Plasma treatment

Plasma treatments were performed in 0.25 mL PCR tubes, and sample volumes were 100 µL. Samples were kept on ice prior to and following plasma treatment, unless otherwise specified, and for treatment were transferred to an in-lab-built Peltier cooling block set to cool to between 2° and 4 °C. All treatment times are listed in methods below.

### L-methionine treatment and analysis

L-methionine (Sigma M9265) was dissolved into 50 mM ammonium acetate (Fisher Chemical A637) at a concentration of 50 µM and treated with a dose curve of plasma from 0 to 60 seconds, with three replicates per timepoint shown. The same stock of ammonium acetate, lacking methionine, was treated in the same fashion for background subtraction during processing. 2.5 µL per sample was directly injected into an ESI-TOF mass spectrometer (Agilent 6210 G1969A) at a flow rate of 40uL/min and a flow buffer of 50:50 acetonitrile:water. Data was collected over the sample injection peak and the corresponding treated background/blank described above. Instrument parameters were as follows: for ESI, voltage was set to 3.5 kV, gas temp was set to 325 °C, drying gas was set to flow at 9.0 l/min, nebulizer was set to 30 psig. For TOF, fragmentor was set to 130 V and skimmer was set to 60 V. For TOF data acquisition in positive mode, scan range was 50–3200 m/z, in profile mode, with 10,000 transients/scan and 0.89 cycles/second. Data was analyzed with Analyst QS (Sciex). Scans were averaged over the injection peak and subsequent treated blank injection per sample, and the treated blank injection peak was used for background subtraction to result in the signal intensity for the peak at m/z 166 as shown. L-methionine sulfoxide (Sigma M1126) was used to build a concentration curve analyzed using the same parameters as listed above. Concentrations and signal intensities are shown in Fig X.

### BSA treatment and sample processing and analysis

BSA (Sigma A7906) was dissolved into PBS (0.1 M sodium phosphate, 0.15Msodium chloride, pH7.2) at a concentration of 50 µM. Samples were treated for 0, 15, 30, or 60 seconds, with three replicates per sample. Following exposure, protein was precipitated with ~10% TCA for 30 minutes on ice and pelleted by centrifugation at 4 °C and 16Kxg for 10 minutes. Supernatant was removed and 100 µL ice cold acetone was added and the solution was vortexed. The sample was pelleted by centrifugation at 4 °C and 16Kxg for 10 minutes. The supernatant was then removed, and the pellet was resolubilized into 8 M Urea in 50 mM ammonium bicarbonate. The sample was diluted to 4 M urea with 50 mM ammonium bicarbonate, reduced with 5 mM DTT for 45 minutes at 50 °C, alkylated with 15 mM IAA for 45 minutes in the dark at room temperature, and then diluted to 1 M urea with 50 mM ammonium bicarbonate. The samples were digested overnight for 16 hours at 37 °C with 1ug of Trypsin (Promega) and 1ug Lys-C (Wako) concurrently.

Following digestion, samples were desalted and concentrated using Omix tips (Agilent) and the following protocol: tips were equilibrated with 100 µL of buffer B (80% acetonitrile, 20% 0.1% formic acid in water), then buffer A (0.1% formic acid in water). The sample was slowly run through the tip five times, then the tip was washed three times with buffer. The sample was then eluted with 100uL buffer B into a fresh tube, and dried down via speed vac. Dried down peptides were resuspended into 80 µL 0.1% formic acid in water, and 1 µL of peptides was injected for spectral analysis (details below).

For the BSA digest experiment, conditions and processing were identical to that above, but an equivalent amount of BSA was digested into peptides, desalted as described, resuspended into PBS, plasma treated, then desalted a second time for spectral analysis.

For SDS-PAGE analysis, shown in Fig. S3, 1uL of treated BSA was loaded onto a Bolt 4–12% Bis Tris Plus Gel (Thermo Scientific), and run in a Bolt Gel Tank (Thermo Scientific) at 160 V for 30 minutes.

### EGFR and EGF reaction and sample treatment

The ecto domain of human epidermal growth factor receptor (residues Met1-Gly645), expressed in human cells (Sino Biological, Inc., 10001-H02H), was solubilized in PBS for a stock concentration of 2.16 µM. This was diluted to ~500 nM for treatment. For reaction with EGF, human EGF expressed in *E. coli* and tested for activity (Sino Biological, Inc., 10605-HNAE) was solubilized in PBS for a stock concentration of 32 µM. A mix of EGFR and EGF was made with a molar ratio of ~3:1 EGF:EGFR (~1.2 µM:430 nM)and reacted for 15 minutes at room temperature with gentle vortexing to mix every few minutes after which samples were aliquoted for treatment.

In triplicate, EGFR alone or EGFR + EGF were treated with plasma for 0, 5 or 10 seconds. Samples were processed identically as described for BSA experiments, except protein was digested with 300ng each of Trypsin and Lys-C, resolubilized into 20 µL of 0.1% formic acid in water, and 2 µL was injected for spectral analysis.

### Peptide mass spectral analysis

The amount of peptides that were injected listed per experiment described above. For the LC (Agilent 1100 series G2226A nanopump) method, buffer A was 0.1% formic acid in water (Fisher Chemical LS118), and buffer B was 0.1% formic acid in acetonitrile (Fisher Chemical LS120). For an overall 80-minute method, peptides were injected onto the column at a flow rate of 500nL/min from min 0–29 in 0% B. The gradient was then ramped to 95% B from min 53–58, then switched to 0% B from min 58–62, and flowed at 0% B at 500nL/min to min 65.

With the above gradient conditions, peptides were injected onto a PepMap RSLC C18, 3 µm, 100 Å, 75µmx15 cm reversed phased analytical column (Thermo Scientific) set to 60 °C and ionized at 1.85 kV via electrospray ionization fed into an Orbitrap Elite mass spectrometer (Thermo Scientific) for analysis. Data-dependent acquisition settings were as follows. From the MS1 spectra acquired at a resolving power of 120 K, from the m/z 350–1800 in profile mode, the top 20 ions were selected for MS/MS analysis, rejecting + 1 charge states. Ions for MS/MS analysis were isolated with a 1.0 m/z isolation width, and fragmented with CID with a normalized collision energy of 35.0, an activation Q of 0.25, and activation time of 10ms. Dynamic exclusion was used, with a repeat count of 2, a repeat duration of 12 s, and an exclusive duration of 12 s. Raw data was uploaded to and made publicly available via the Chorus project (https://chorusproject.org).

Raw data was searched using Proteome Discoverer 2.1 (Thermo Scientific) and the Mascot search algorithm (Matrix Sciences). A database consisting of common contaminants with the proteins of interest was used for searching. Trypsin was specified as the protease, with 2 missed cleavages allowed. Ten ppm tolerance was used for the precursor mass tolerance, and 0.6 Da was used for the fragment mass tolerance. All modifications were set to variable. These included deamidation (NQ), carbamidomethylation (C), and oxidation (FHIKLMPRTVWYACDENQS). Data was automatically filtered by PD to a 1.0% strict max FDR, and area under precursor ion chromatograms is calculated by PD.

Data was output to Excel (Microsoft) for further manual processing. The output was filtered with the following conditions: protein of interest, confidence = high, rank and search engine rank = 1, and ion score ≥20. The oxidation events per PSM were then counted in a separate column, and the data was filtered for redundancy based on the data in total and modification count, rather than individual modification events. Note that this removes extracted ion chromatograms (XICs), which result from multiple PSMs under the same peak and prevents redundant summation while allowing multiple charge states to be considered in the summation. Next, areas under the XICs were summed per peptide for unmodified variants and modified variants, and percent modification was calculated based on this. These numbers make the basis for average and standard-deviation calculations shown in the tables and figures, as well as for the statistical tests performed. All statistical tests were two-tailed and unpaired with equal variance specified. To examine residues of modification, manual examination of the dataset was performed. Graphs and figures were made using Excel and Illustrator (Adobe), and crystal structures were obtained from the PDB archive. Crystal structure images were created using Pymol (Schrodinger).

## Electronic supplementary material

Supplementary Dataset 1

Supplementary Dataset 2

Supplementary Dataset 3
