# Supplementary material for: Plasma-Generated OH Radical Production for Analyzing Three-Dimensional Structure in Protein Therapeutics
Source: Sci Rep. 2017 Oct 11;7:12946. doi: 10.1038/s41598-017-13371-7 (PMC5636892; doi:10.1038/s41598-017-13371-7)
Supplement: Supplementary file 1 — Supplementary Dataset 1 [file 41598_2017_13371_MOESM1_ESM.doc]

**Supplemental Data for:**

Plasma-Generated OH Radical Production for Analyzing Three-Dimensional Structure in Protein Therapeutics

Benjamin B. Minkoff^1^, Joshua M. Blatz^2^, Faraz A. Choudhury^2^, Daniel Benjamin^2^, J. Leon Shohet^2^, Michael R. Sussman^1^*

^1^Department of Biochemistry, University of Wisconsin-Madison, Madison, WI

^2^Department of Electrical and Computer Engineering, University of Wisconsin-Madison, Madison, WI

* To whom correspondence should be addressed: msussman@wisc.edu

**Supplemental Figure Captions**

**Figure S1.** A, Data collected from ESI-TOF analysis of methionine treated with PLIMB. Signal intensity are arbitrary units, and for each data point, n=3 and error bars are ± standard deviation. B, Concentration curve of pure methionine sulfoxide.

**Figure S2.** Example data for conversion of methionine (m/z 150) to methionine sulfoxide (m/z 166) spanning the range of treatment times.

**Figure S3.** Gel demonstrating that throughout exposure, BSA remains intact. 1µL/3µg was loaded onto gel.

**Figure S4.** Complete set of oxidation data for BSA, scaled to A, 100%, B, 10%, and C, 1%. For each bar, n=3 and error bars are ± standard deviation.

**Figure S5.** Complete set of oxidation for untreated EGFR and EGFR+EGF samples, scaled to 100%, 20%, and 2%. For each bar, n=3 and error bars are ± standard deviation.

**Figure S6.** Complete set of oxidation for five second PLIMB treated EGFR and EGFR+EGF samples, scaled to 100%, 20%, and 2%. For each bar, n=3 and error bars are ± standard deviation.

**Figure S7.** Complete set of oxidation for ten second PLIMB treated EGFR and EGFR+EGF samples, scaled to 100%, 20%, and 2%. For each bar, n=3 and error bars are ± standard deviation.

**Figure S8.** Crystal Structure of inactivated EGFR. Domain I is shown in red, Domain II green, Domain III blue, and Domain IV orange. Residues identified as decreasing in oxidation, in a statistically-significant fashion, with EGF added, are highlighted yellow. Image of 1NQL (reference 18) created using PyMOL (The PyMOL Molecular Graphics system, Version 1.8, Schrödinger, LLC).

**Supplemental Table Captions**

**Table S1.** Modification percentages in native and denatured BSA in control and PLIMB-treated samples. For each data point, n=3 and error bars are ± standard deviation.

**Table S2.** Modification percentages in EGFR alone and EGFR with added EGF in control and PLIMB-treated samples. For each data point, n=3 and error bars are ± standard deviation.

Figure S1

Figure S2

Figure S3

Figure S4

Figure S5

Figure S6

Figure S7

Figure S8


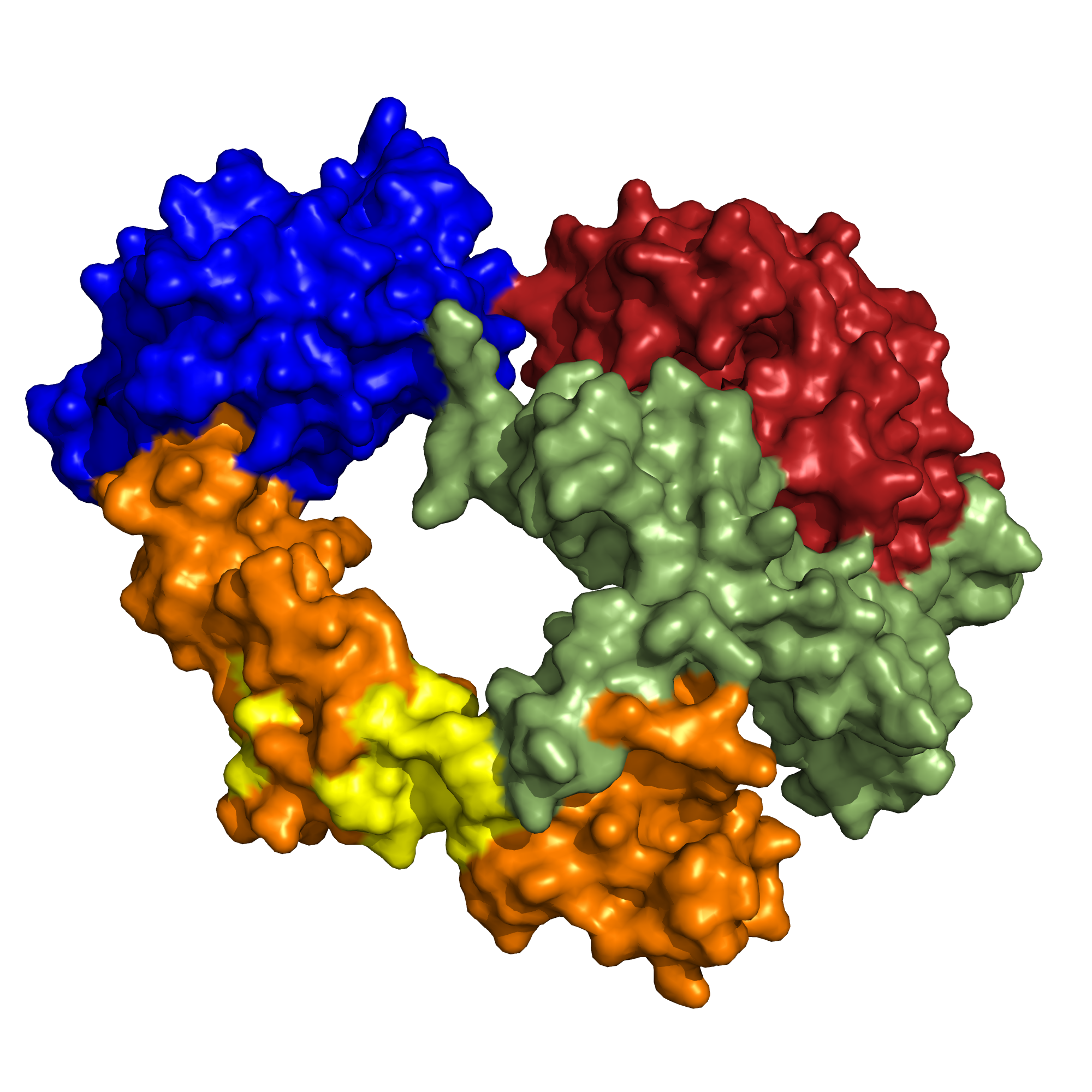


Table S1

Table S2
